# Optimum Choice of Randomly Oriented Carbon Nanotube Networks for UV-Assisted Gas Sensing Applications

**DOI:** 10.1021/acssensors.3c01185

**Published:** 2023-09-08

**Authors:** Katarzyna Drozdowska, Adil Rehman, Janusz Smulko, Aleksandra Krajewska, Bartłomiej Stonio, Pavlo Sai, Aleksandra Przewłoka, Maciej Filipiak, Krystian Pavłov, Grzegorz Cywiński, Dmitry V. Lyubchenko, Sergey Rumyantsev

**Affiliations:** † Department of Metrology and Optoelectronics, Faculty of Electronics, Telecommunications, and Informatics, Gdańsk University of Technology, G. Narutowicza 11/12, Gdańsk 80-233, Poland; ‡ CENTERA Laboratories, Institute of High Pressure Physics PAS, Warsaw 01-142, Poland; § Centre for Advanced Materials and Technologies CEZAMAT, Warsaw University of Technology, Warsaw 02-822, Poland; ∥ Institute of Optoelectronics, Military University of Technology, gen. Sylwestra Kaliskiego 2, Warsaw 00-908, Poland; ⊥ Division of Micro and Nanosystems, KTH Royal Institute of Technology, Malvinas Väg 10, Stockholm SE-100 44, Sweden

**Keywords:** carbon nanotubes, low-frequency noise, UV irradiation, gas sensor, biocontrol, ethanol

## Abstract

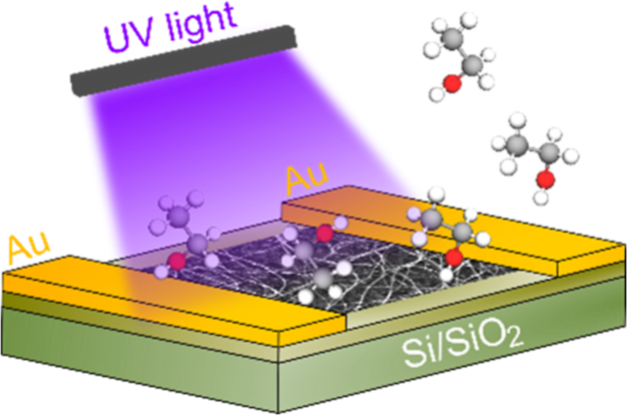

We investigated the noise and photoresponse characteristics of various optical transparencies of nanotube networks to identify an optimal randomly oriented network of carbon nanotube (CNT)-based devices for UV-assisted gas sensing applications. Our investigation reveals that all of the studied devices demonstrate negative photoconductivity upon exposure to UV light. Our studies confirm the effect of UV irradiation on the electrical properties of CNT networks and the increased photoresponse with decreasing UV light wavelength. We also extend our analysis to explore the low-frequency noise properties of different nanotube network transparencies. Our findings indicate that devices with higher nanotube network transparencies exhibit lower noise levels. We conduct additional measurements of noise and resistance in an ethanol and acetone gas environment, demonstrating the high sensitivity of higher-transparent (lower-density) nanotube networks. Overall, our results indicate that lower-density nanotube networks hold significant promise as a viable choice for UV-assisted gas sensing applications.

Carbon nanotubes (CNTs) have been extensively used in various applications owing to their exceptional electrical, mechanical, and optical properties (^1,2^ and references therein). The ultralarge surface-to-volume ratio of nanotubes makes them an appealing candidate for sensing applications.^3^ They can also be used as effective chemical and biological sensors (^4,5^ and references therein) owing to their high adsorption capacity that can detect the interaction of even a single molecule on their surface. The ultrafast response of this sp^2^-hybridized network of carbon atoms plays a significant role as an optical sensor.^6^ CNTs are also an excellent alternative to traditional carbon electrodes and are considered promising for electrochemical-based sensing devices.^7–11^ They also exhibit excellent surface sensitivity and, therefore, have significant potential for gas sensing applications (^12,13^ and references therein). It is also well-known that the sensitivity of the sensors can be increased upon ultraviolet (UV) light illumination,^14–16^ which in turn boosts the adsorption/desorption processes on the active surface layer and enhances the detection mechanism of gas molecules. UV illumination also reduces the recovery time and drift, the essential parameters for effective sensing devices.^17^ The spectral response of CNTs to irradiation from UV-C to infrared (IR) was studied in refs (18) and (19). It was found that shorter wavelengths affect the electrical properties of CNTs more significantly.^18^ Denser nanotube networks were observed to exhibit greater absorbance but a lower resistive response to the incoming irradiation.^19^

The sensitivity of the sensors can be further enhanced by utilizing the power spectral density of resistance, current, or voltage fluctuations at low frequencies, where 1/*f* noise dominates.^16,20–22^ Noise is one of the essential parameters of electronic devices, which can limit the sensors’ sensitivity. However, the specific features (e.g., changes in the slope of the noise spectra induced by Lorentzian components), together with resistive responses, can also be used as sensing parameters.^23,24^

In several cases, low-frequency fluctuations are more sensitive to gas molecules than the DC resistance of the device itself. Various reports demonstrated low-frequency noise measurements to examine the response characteristics of the sensors.^25–28^ For example, it was observed for CNTs that their noise level decreases with increasing concentrations of nitrogen dioxide (NO_2_), with sensing profiles accelerated by UV light (365 or 275 nm).^16^ Unlike the DC resistance measurements, noise spectroscopy gives a stochastic fingerprint of the gas molecules interacting with the sensing surface.^29^ The gas-vapor-induced Lorentzian noise serves as a unique signature of the particular characteristic frequency of a given gas molecule.^30,31^

Previously, many nanotube-based devices have been proposed for sensing applications. However, in counterpart to single or aligned nanotube bundles, randomly oriented CNT network-based devices offer ease of fabrication technology and therefore have significant potential to fabricate cost-effective and efficient sensing devices on a large scale. In this work, we study the network density-dependent photoresponse of CNT-based devices over a wide range of UV light wavelengths ranging from 265 to 375 nm and demonstrate that the device with a smaller density of nanotube networks exhibits a more intense photoresponse to incoming UV irradiation. The study is further extended to the noise properties as well, and it is shown that the device with a less-dense network of CNTs exhibits low noise levels. These results implied that less-dense networks of CNTs are promising candidates for UV-assisted gas sensing applications owing to their low noise level and high sensitivity to incoming UV irradiation. These results are further confirmed by measuring the resistive response and noise of devices with low- and high-density networks of CNTs in ethanol and acetone atmospheres.

## Results and Discussion

### Structural Characterization of the Nanotube Networks

**Figure 1:**
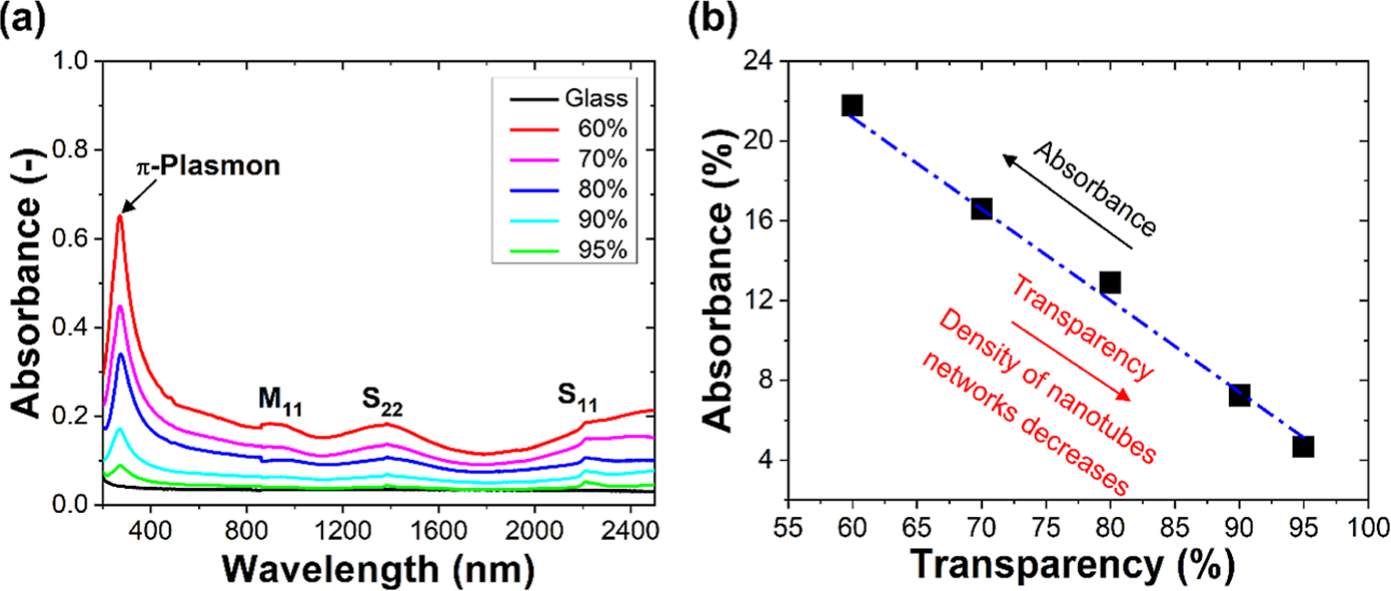
(a) Optical absorbance spectra of nanotube networks of different transparencies (60–95%) with a reference spectrum for glass substrates and (b) absorbance at 555 nm as a function of CNT network transparency. The wavelength of 555 nm was chosen as a human vision peak sensitivity point (corresponding to green light). CNT networks deposited on glass substrates were used for the optical property studies. The labels 60, 70, 80, 90, and 95% show the transparency of nanotube networks in the visible range (particularly at 555 nm).

The optical absorbance spectra of different densities of nanotube networks on glass substrates over a wide range of wavelengths are shown in Figure 1a. For reference, the absorbance spectrum of a glass substrate is also shown here. Each spectrum corresponds to the different densities of nanotube networks and exhibits different transparency in the visible spectrum. The absorbance can be related to the transparency of nanotube networks as it is directly related to the optical transmittance. By using a relation: absorbance = −log(*T*/100), where *T* is the sample transmittance in %. As seen, the lower absorbance corresponds to the higher transparency of the nanotube networks. For the sake of simplicity, we labeled each nanotube network in Figure 1a as 60, 70, 80, 90, and 95%. These labels show the transparency of nanotube networks in the visible range (particularly at 555 nm). One might notice that the sample with the lowest transparency (i.e., 60%) exhibits the highest absorbance and has a more dense network of CNTs in comparison to the sample with the highest transparency (i.e., 95%), which demonstrates the lowest absorbance and has a less-dense network of nanotubes. Figure 1b shows the absorbance of the studied nanotube networks as a function of transparency. The devices with different transparency or absorbances exhibit different densities of nanotube networks. One might also notice the appearance of different peaks in Figure 1a in the vicinity of ∼270, ∼ 960, ∼ 1500, and ∼2200 nm that are referred to as π-plasmons, M11, S22, and S11 peaks, respectively, and are typical for CNTs.

**Figure 2:**
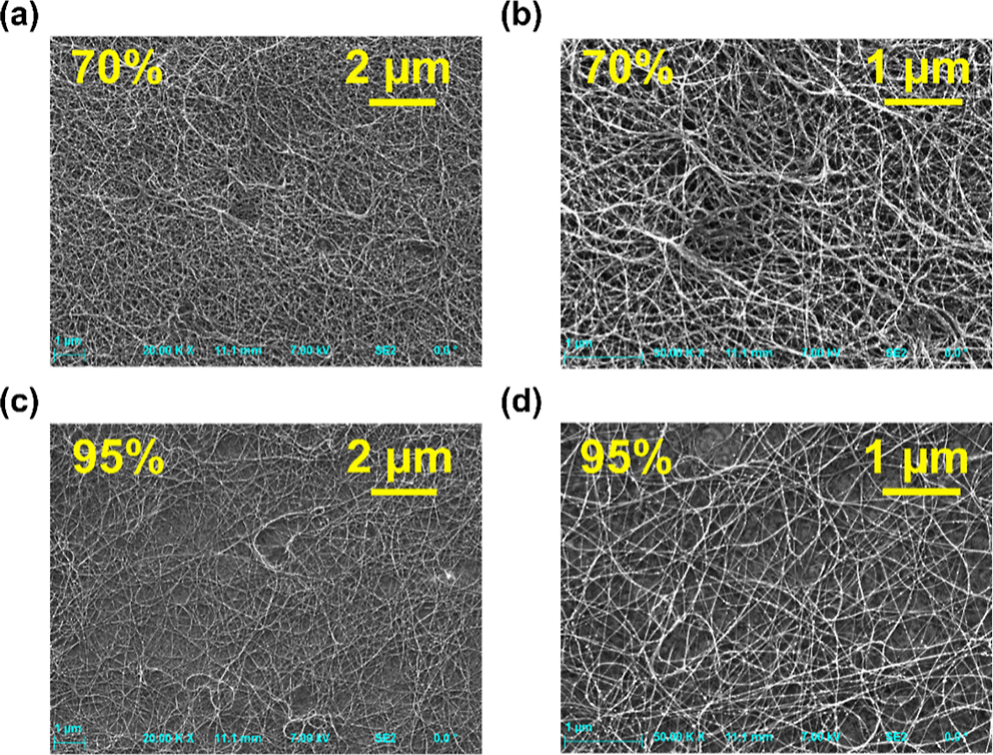
(a–d) SEM images of the nanotube networks of 70% (a,b) and 95% transparency (c,d) in two different resolutions, showing the greater density of the CNT network for the sample of lower transparency.

**Figure 3:**
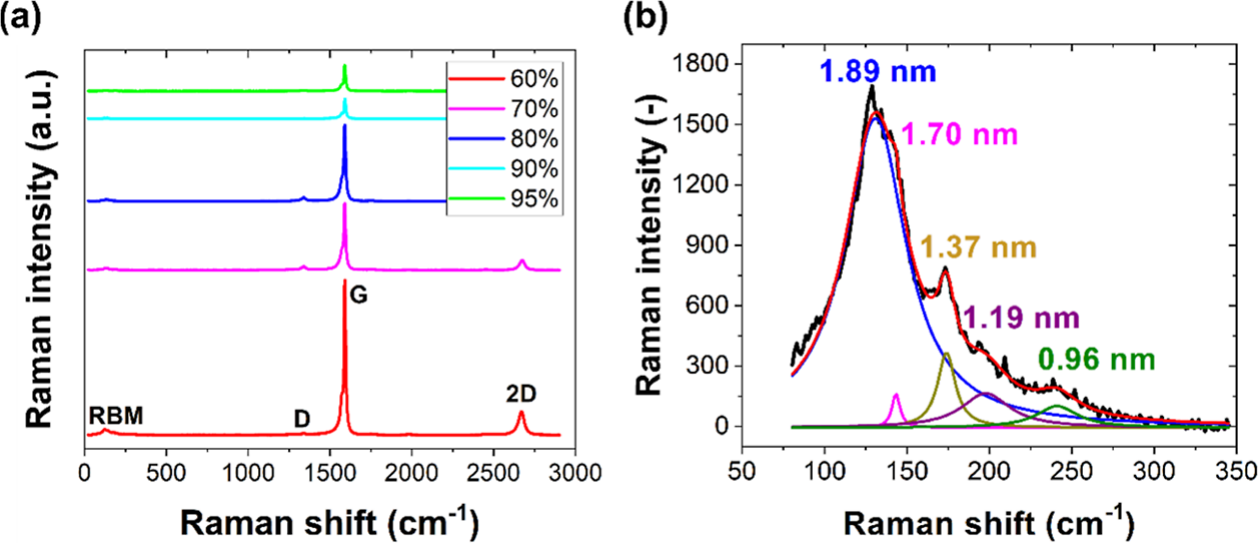
(a) Raman spectra of CNT networks of different transparencies (60–95%) and (b) deconvolution of the Raman spectrum of an exemplary sample of 80% transparency with an estimated diameter of CNTs in the network. For comparison, for networks of 60, 70, 80, 90, and 95% transparency, the main peak corresponds to CNTs with diameters of 2.02, 1.76, 1.89, 1.82, and 1.98 nm, respectively.

The density of nanotube networks and structural morphology were studied via high-resolution SEM. Figure 2 shows SEM images of the 70 and 95% nanotube networks at two resolutions. As seen, the density of the nanotube network in the sample of 70% transparency is greater than the network of 95% transparency. Such observation correlates well with the higher optical absorbance demonstrated by the sample of 70% compared to 95% transparency. The SEM images of other investigated CNT networks can be found in the Supporting Information (Figure S1). Figure 3a shows an example of the Raman spectra of a few studied nanotube networks.

Raman spectroscopy is an effective and nondestructive technique to characterize the properties of low-dimensional materials, including CNTs. The Raman spectrum consists of four peaks. The peak at the lower wavenumber (i.e., ∼ 130 cm^–1^) is called the Raman RBM peak and appeared due to the coherent vibrations of the carbon atoms in the radial direction. This peak is considered a unique feature of the Raman spectrum of CNTs. It also provides information about the diameter *d* of nanotubes via the relation *d* = *A*_0_/(ω_RBM_ – *B*) (here, *A*_0_ = 217.8 cm^–1^, *B* = 15.7 cm^–1^, and ω_RBM_ is the peak frequency in cm^–1^). Figure 3b shows the deconvolution of the Raman RBM peak with Lorentzian curves for one of the exemplary nanotube samples. The main peak corresponds to a diameter of 1.89 nm. The shapes of the peaks also indicate the presence of nanotubes with smaller diameters. Analogous RBM peak deconvolution was performed for the other samples. For samples of 60, 70, 90, and 95% transparency, the main peak corresponded to CNTs of 2.02, 1.76, 1.82, and 1.98 nm diameter, respectively. The number of nanotubes of smaller diameters was found to depend on the specific location of the network. The peak in the vicinity of ∼1340 cm^–1^ is called the Raman D-peak and is associated with the defects in nanotubes, whereas the peak in the vicinity of ∼1590 cm^–1^ is called the graphitic G peak. The ratio of these two peaks can be used to estimate the quality of nanotubes.

### Photoresponse Measurements

**Figure 4:**
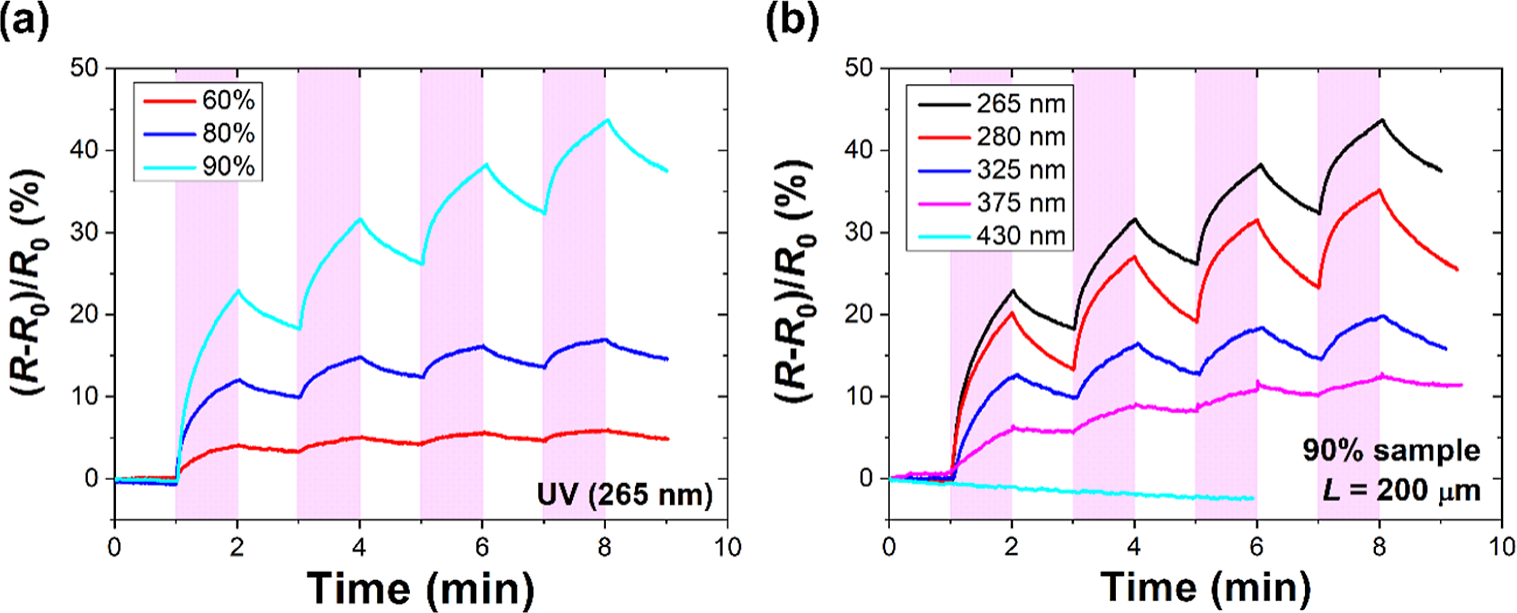
(a) Short-time photoresponse of the CNT networks of different transparencies under UV light at 265 nm showing higher responses of a low-density network (90%) and (b) photoresponses of the sample with 90% transparency (channel length *L* = 200 μm) at various UV-light wavelengths (265 nm −430 nm). Shadowed regions refer to 1 min illumination cycles. *R*_0_ denotes baseline resistance at a time of 0 min.

The photoresponse experiments were performed by employing a semiconductor parametric analyzer. Figure 4a shows the relative changes in the resistance of a few studied densities of CNT networks under short cycles of UV irradiation. As seen, the resistance increases for all investigated samples upon UV illumination (i.e., negative photoconductivity), owing to the desorption of gas molecules attached to the surface of CNTs. A more detailed description of the UV light effect of CNT-based device resistance can be found in refs (16), (32), and (33). Interestingly, the sample with a less-dense network of CNTs (in this case, 90% transparency) exhibits a higher response to incoming UV irradiation than the sample with a higher network density (i.e., 60% transparency). The same effect was previously observed for single-walled CNT films of different densities.^19^ This signifies that less-dense networks of CNT-based devices are more sensitive to incoming UV irradiation and could be a promising candidate for low-cost UV light-activated gas sensing applications. Figure S2 shows the longer cycle of UV illumination (20 min) and the relative change in the resistance of two exemplary samples of CNTs with differently dense networks. One might also notice in Figure S2 that the resistance dependences on time are slow and nonexponential (i.e., the longer the time, the higher the characteristic time of the resistance change). This feature is common to the 1/*f* noise spectrum, implying a wide range of relaxation times. The resistance returns to its initial value after a few hours in the dark. For less-dense networks of CNTs, the short wavelength of UV irradiation reaches relatively more of a specific active area of nanotubes than in dense networks. This implies that activation of the CNTs’ surface and generation of charge carriers within the network can be more efficient in low-density layers. Figure 4b shows the relative change in the resistance of one exemplary device (90% transparency) under short cycles of UV illumination of different wavelengths in reference to the resistance in the dark (*R*_0_). The device’s response to visible light (430 nm) is also demonstrated for comparison. The device undergoes a continuous decrease in DC resistance upon irradiation with visible light.

The slow heating of the CNT network and humidity in the ambient air are factors responsible for the drift in the resistance of the studied device.^33–35^ Furthermore, the energy carried by visible light is insufficient to increase the concentration of photogenerated carriers, which in turn boosts the adsorption/desorption processes. However, on the other hand, UV irradiation increases the concentration of photogenerated carriers and enhances the adsorption/desorption processes. The irradiation of the device with UV light of a shorter wavelength (265 nm) causes a more significant change in the relative resistance of the device compared to UV irradiation of longer wavelengths owing to the higher energy provided to the sample surface. Afterward, the change in resistance is limited by the slow dynamics of oxygen and water desorption on the surface of nanotubes. Our observations agree with the effect of short UV light wavelengths, as demonstrated before, for CNT-based UV light detectors.^18^

Additionally, we investigated the effect of humidity on the CNT network by comparing baseline resistances in laboratory air (indoor air of relative humidity, usually in the range of 40–60% depending on the day) and dry synthetic air (S.A.). We observed a constant drift downward in sensor resistance after applying a voltage bias of 0.1 V in ambient air and an increase in resistance of between 3 and 5% for the introduction of S.A. (after saturation for 30 min). Under UV light, this effect was even magnified. For the 70% sample, the response to irradiation increased around three times more in S.A. than in laboratory air and around two times more for the 90% sample. Based on these observations, we conclude that UV light facilitates humidity species desorption and cleans/prepares the CNTs’ surface for molecular adsorption of target gases.

### Low-Frequency Noise Measurements

Noise is one of the most critical parameters and can limit the sensitivity of any electronic device. It is well-known that randomly oriented networks of CNT-based devices have considerable potential for cost-effective UV-assisted gas sensor application. However, the noise level of nanotube networks of various densities can be different and may reduce the sensitivity of such sensors and compromise their performance. Therefore, we extended our investigation of nanotube networks and measured the low-frequency noise characteristics for the exemplary samples.

**Figure 5:**
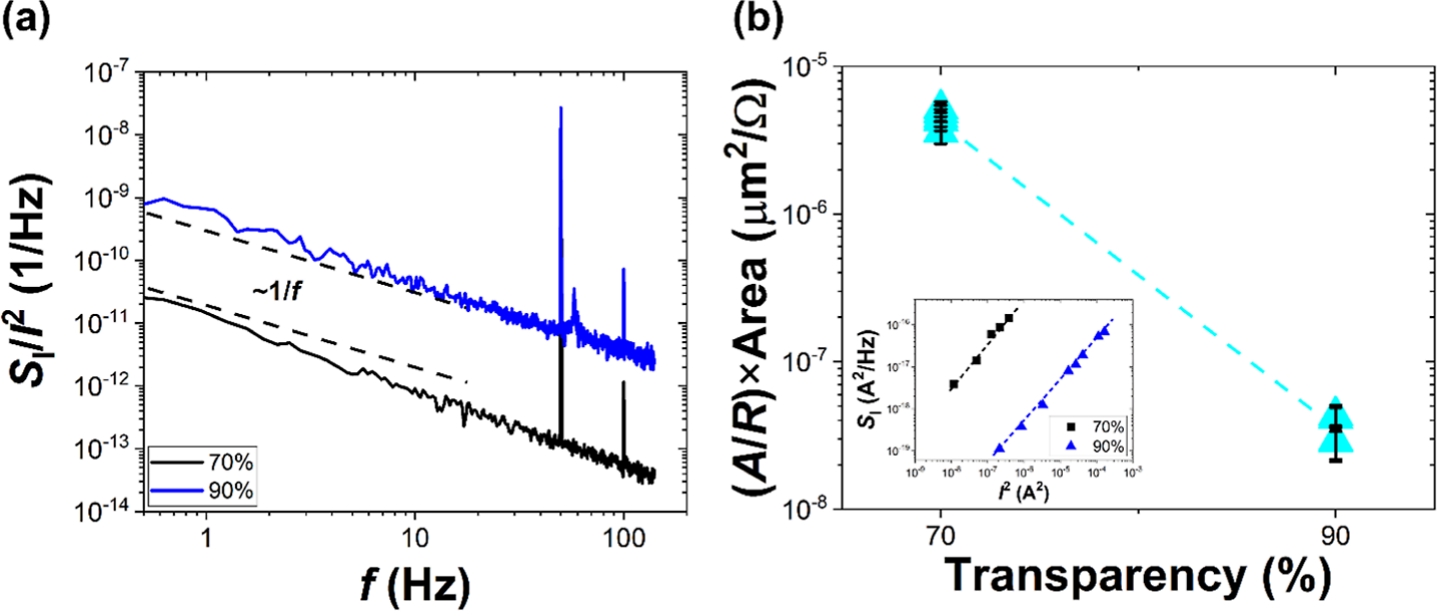
(a) Power spectral density of current fluctuations *S*_I_(*f*) normalized to current squared *I*^2^ showing 1/*f* noise for two samples chosen for gas sensing experiments (70 and 90% transparency). Dashed lines correspond to a 1/*f* noise shape preserved regardless of the sample’s transparency. (b) Noise amplitude *A* normalized to sample resistance *R* and area for two devices with the inset presenting *S*_I_(*f*) as a function of current squared *I*^2^ for devices selected for further gas sensing experiments. Normalized noise amplitude *A* was calculated based on five measurements for each sample as *S*_I_(*f*)/*I*^2^ × *f* at *f* = 10 Hz. Black error bars indicate the standard deviation calculated from five independent measurements, showing device-to-device reproducibility.

Figure 5a shows the noise spectra of two different densities of CNT-based samples. Both samples were chosen for further gas sensing experiments employing resistive and noise studies, with a 70% device representing the high-density networks and a 90% device representing the low-density networks. As seen, the samples of 70 and 90% transparency exhibit 1/*f*-like noise spectra. The visible spikes at 50 Hz represent the frequencies of the European power grid. The dependence of *S*_I_ as a function of the square of the current for both samples is shown in the inset in Figure 5b. The power spectral density of current fluctuations is proportional to the square of the current, which implies that the resistance fluctuations are responsible for the origin of the 1/*f* noise. Figure 5b shows the noise levels of two different densities of the nanotube network chosen for further experiments. The *y*-axis is intentionally normalized to the resistance and area of the nanotube to compare the noise level of different densities of the nanotube network. The resistance of the studied devices is shown in Figure S3. As seen from Figure 5b, the sample with higher transparency (i.e., a less-dense network of nanotubes) exhibits a lower noise level, potentially making it an appropriate candidate for sensing applications. At the same time, the less-dense networks of the nanotube sample were reported to interact strongly with the incoming UV light, which, detection-wise, can be advantageous (see Figures 4 and S2 for UV light-activated responses).

### Gas Sensing Experiments

**Figure 6:**
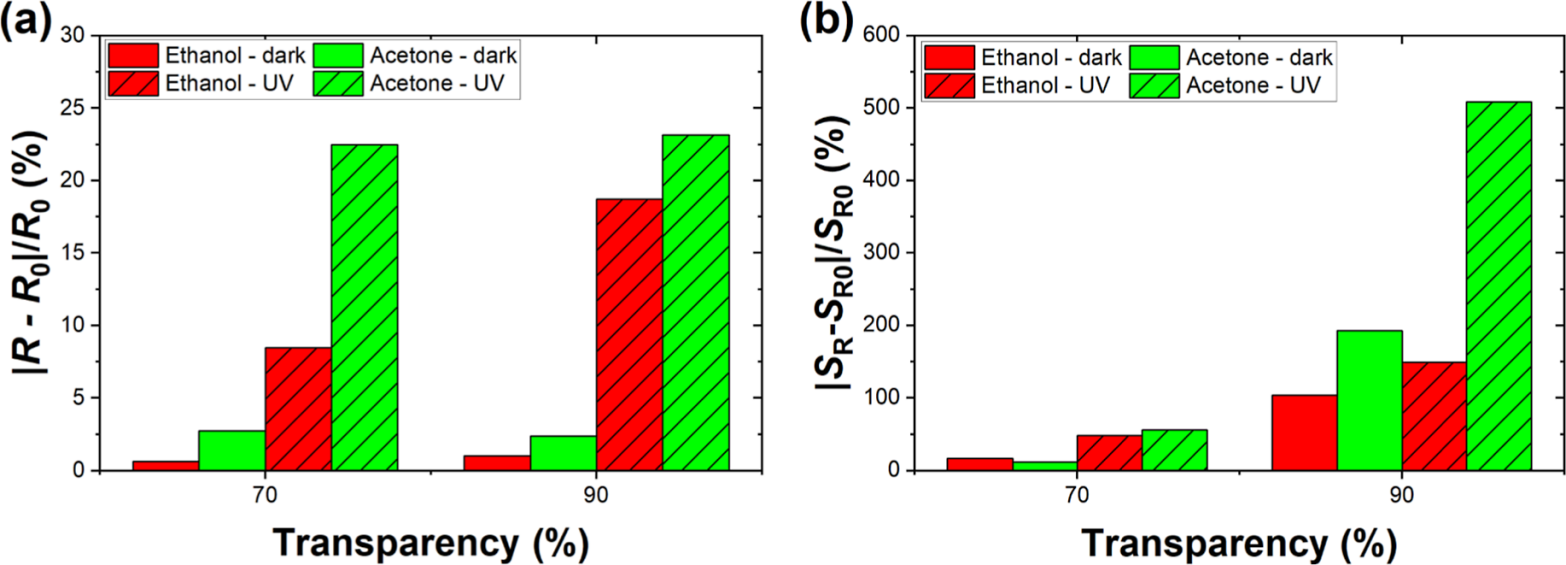
(a) Relative change in sensor resistance *R* and (b) power spectral density of resistance fluctuations *S*_R_ normalized to sensor resistance squared *R*^2^ at *f*_0_ = 0.5 Hz for ethanol (140 ppm) and acetone (110 ppm) in the dark and under UV 275 nm irradiation for samples of 70 and 90% transparency. Reference resistance *R*_0_ and reference power spectral density *S*_R0_ designate S.A. conditions.

To confirm that low-density networks of CNTs can improve gas detection more significantly than high-density networks for UV-assisted sensing applications, the resistance and low-frequency noise of two samples of different densities of networks of CNTs were measured under ethanol and acetone vapor. We specifically chose sensors with a CNT network of 70% transparency (representing a high-density sample with a lower response to UV light) and 90% transparency (representing a low-density network with a higher resistive response to UV light). Additionally, the 90% sample produced more reproducible responses than the 95% sample because of high randomness and local differences in the network’s structure while still maintaining a high response to UV light [only a 2-percentage point difference in UV light (275 nm) response between the 90 and 95% samples]. The experiments were remeasured under UV light to prove that UV irradiation can further enhance the sensing characteristics of the sensors. Figure 6a shows the relative change in the resistance of two differently dense CNT networks (i.e., 70 and 90% transparencies) under an ethanol and acetone vapor atmosphere.

As seen, the sample with a less-dense network of CNTs (i.e., 90% transparency) exhibits higher changes in resistance for the detection of both gases owing to the more porous network and thinner layer with multiple binding sites that actively participate in the surface adsorption/desorption processes during molecular detection. Moreover, the UV light further enhances the resistive response at least several times for each sample of different densities of CNT networks. The high UV light absorption for less-dense CNT networks correlates well with the high sensitivity toward gas molecules regarding resistive responses. Figure 6b shows the relative change in the resistance fluctuations of two different dense networks of CNT samples (i.e., 70 and 90% transparencies) under an ethanol and acetone vapor atmosphere. Similarly to resistive responses, UV light tends to increase the relative change in the sensor’s noise for both investigated samples of different densities of CNT networks. One might notice that the relative change in the noise is higher than in the resistance of samples (around eight times for ethanol and around 22 times for acetone under UV light) and agrees with previous results of fluctuation-enhanced gas sensing by CNT networks.^16^Figure S4 shows an example of noise spectra for one of the studied devices under S.A., ethanol, and acetone atmospheres. These findings demonstrate the potential of low-density CNT networks for cost-effective gas sensing applications and show that sensing characteristics can be further enhanced by employing UV light to improve the adsorption/desorption processes during molecular detection.

**Figure 7:**
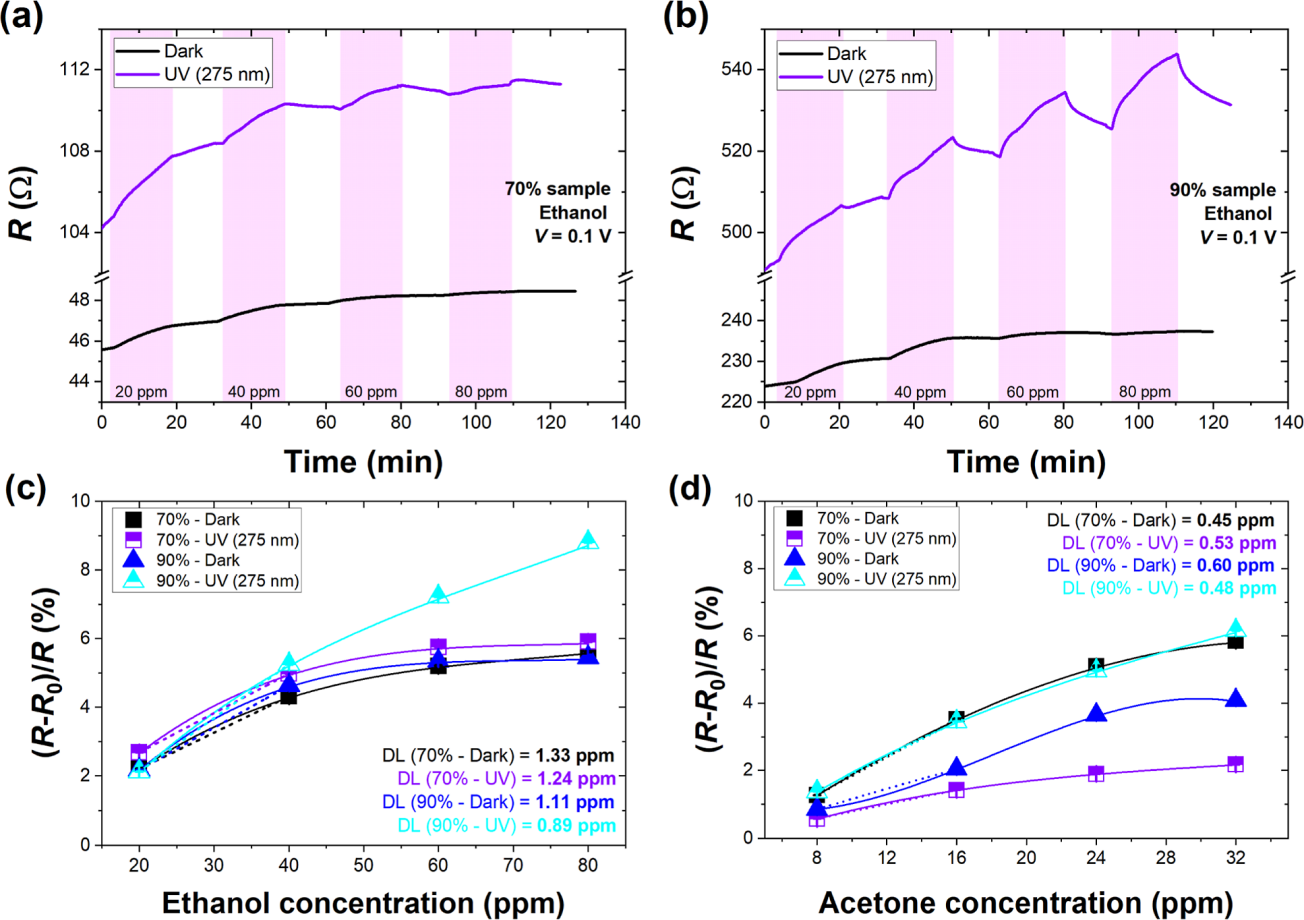
Time–domain studies for four cycles of ethanol introduction (concentrations 20–80 ppm) in the dark and under UV light (275 nm) for CNT network sensor of (a) 70% transparency and (b) 90% transparency. Relative changes in sensor resistance *R* for all investigated conditions were (c) ethanol and (d) acetone. Estimated DL values are depicted on the graphs. Reference resistance *R*_0_ designates S.A. conditions before the first cycle of the target gas introduction. Voltage bias was set to 0.1 V for all time-response measurements. Vertical markers presented with data points in (c,d) designate error bars associated with the accuracy of the measurement system.

Since a low-density network (90% transparency) under UV irradiation was observed to enhance sensor responses to ethanol and acetone, additional resistive measurements were conducted to provide a more comprehensive view of the sensors’ performance. Figure 7a,b presents the time–domain curves for selected ethanol concentrations (20–80 ppm) for 70 and 90% of samples in the dark and under UV light (275 nm). The analogous time–domain curves for acetone are depicted in Figure S5. As expected, the resistive responses in the ethanol atmosphere are more pronounced under UV light for both investigated samples. At the same time, we observe the short-term drift, especially in the first cycle of introducing the lowest concentration of ethanol (20 ppm). In the dark, the recovery is feeble. Under irradiation, the recovery rate is visibly improved but insufficient to obtain the baseline during 15 min.

Based on the relative changes in sensor resistance, we estimated the detection limit (DL) for all investigated conditions (see Methods for the description of the DL estimation procedure). The values are depicted in Figure 7c for ethanol and Figure 7d for acetone. In the case of ethanol, only for 90% sample, and under UV light, DL was reduced below 1 ppm. For the 70% sample, UV light only slightly improves DL, but in both cases, the values are higher than for the 90% sample, suggesting enhanced detection for a low-density network and UV light. We observe that UV irradiation increases the sensors’ sensitivity to ethanol and accelerates the recovery. Additionally, a low-density network interacting strongly with UV light is observed to improve the sensor’s performance. DL estimated for a UV-assisted 90% sample of 0.89 ppm is lower than for sensors based on single-walled CNTs ranging from 5 to 50 ppm reviewed in other works and usually utilizing doping or surface modifications with metals and metal oxides.^13^ Thus, we observe the potential of CNT networks of higher transparency fabricated via a simple method to be highly sensitive ethanol sensors under UV irradiation. Interestingly, for acetone, UV light improves the DL only for 90% of the sample, reaching 0.48 ppm. The unexpectedly low DL for the 70% sample and dark conditions might result from relatively high changes in sensor resistance accompanied by constant drift, especially in the first cycle of target gas introduction (see Figure S5). Since organic molecules are usually weakly bonded to the carbon surfaces, we believe that such a low acetone concentration may not overcome sensor drift to produce a reliable response.

## Conclusions

To conclude, the structural, photoresponse, and noise characteristics of different densities of randomly oriented networks of CNT-based sensors were investigated. All of the investigated sensors manifested negative photoconductivity upon UV irradiation. It is also shown that less-dense networks of CNT sensors exhibited a higher change in DC resistance upon UV irradiation. The low-frequency noise measurements revealed that all studied sensors exhibit a 1/*f* noise shape with noise levels depending on the densities of nanotube networks. Further normalization of the noise to the DC resistance and active area of the sensors showed that noise decreases as the transparency of the nanotube network increases (i.e., the density of the nanotube network decreases). These results showed the potential advantage of low-density networks of CNT-based devices for UV-assisted gas sensing applications. Further measurements of the noise and DC resistance in the ambiance of ethanol and acetone vapors confirmed that less-dense CNT networks interact strongly with gas molecules and the sensing capabilities can be further enhanced by employing UV light irradiation. We want to highlight that detailed measurements with different target gases provide a more comprehensive view of whether the optimum choice differs for various vapors. Nevertheless, our findings confirm that low-density networks enhance resistive and noise responses for light-assisted ethanol sensing. For acetone detection, UV light enhances the response of the 90%-transparent sensor. However, the intense time drift (especially in the dark) prevents straightforward conclusions for low acetone concentrations and requires a more thorough investigation.

## Methods

The randomly oriented CNT networks (single-walled CNTs, SWCNTs) were synthesized via an aerosol chemical deposition technique and precipitated on a nitrocellulose substrate with the density dependent on the process time. The nanotube networks were then transferred onto an oxidized silicon substrate (with predeposited gold electrodes) using the dry transfer method. The detailed device fabrication and nanotube transfer process can be found elsewhere.^34^ The densities of the studied nanotubes were examined using a UV–vis–NIR LAMBDA 1050 spectrophotometer and scanning electron microscope (SEM). We want to notice that we associate the density of CNTs in the networks with their optical transparency based on optical and SEM imaging. Thus, the higher transparency of the sample is associated with a lower density of the CNT network. A Renishaw inVia Raman microscope was employed to estimate the mean diameter of CNTs. The excitation power of the Nd/YAG laser beam (532 nm) was 2.1 mW, which was directed to the sample through a 100× objective lens.

All of the measurements were conducted at room temperature and ambient pressure. The effect of irradiation over a wide range of wavelengths on different densities of the nanotube networks was studied with a semiconductor parametric analyzer. The light sources were bought from EPIGAP and ProLight Opto^36,37^ and positioned approximately 1 cm from the nanotube surface. The low-frequency noise was measured by recording voltage fluctuations across the load resistor (*R*_L_), connected in series with the device of resistance *R*_0_. The recorded signal was amplified with a low-noise voltage amplifier (Signal Recovery MODEL 5184) and fast Fourier transform by a photon dynamic signal analyzer. Later on, the power spectrum of voltage fluctuation (*S*_V_) was converted into current fluctuations (*S*_I_) by using the equation *S*_I_ = *S*_V_((*R*_L_ + *R*_0_)/(*R*_L_*R_0_))^2^.^38^ The background noise of the system was estimated by replacing the device with a metal resistor of the same value as *R*_0_. In our case, the observed background noise of the system was at least 20 dB smaller than the noise of the studied devices.

Ethanol and acetone vapors were chosen as exemplary target gases for the gas sensing experiments. The measurements were performed by keeping the devices inside a metal shielding box to avoid external electromagnetic interference. Target vapor was produced by transferring dry S.A. through a glass bubbler filled with liquid ethanol or acetone. A flow of 50 mL/min determined ∼140 ppm ethanol and ∼110 ppm acetone in the vicinity of the sensing surface, and the sample surface was exposed to the target gas via a metal pipe connected to the gas distribution system. The gas pipe was positioned within 0.5 cm of the sample surface and a mass flow controller (Analyst-MTC, GFC17) regulated the constant overall gas flow. Time–domain studies were performed using the calibration gases ethanol and acetone. Mixing target gases with S.A. at specific proportions enabled obtaining 20–80 ppm ethanol and 8–32 ppm acetone. The voltage bias was set to 0.1 V for all resistive measurements. The theoretical DL was calculated based on relative changes in sensor resistance under selected conditions. A third-order polynomial function was fitted to the experimental data points. The deviation between experimental and theoretical values of the sensor response was used to estimate the root-mean-square (rms) value. Additionally, a linear fit was performed for the quasilinear region of the sensor responses observed at low concentrations of target gases. Based on the calculated rms and slope (from the linear fitted function), the DL was determined according to the formula: DL = (S/N)·rms/slope, where S/N refers to a signal-to-noise ratio equal to 3. For noise response studies, *S*_V_ was converted into resistance fluctuations *S*_R_ via the equation *S*_R_ = *S*_V_/*I*^2^, where *I* is the current flowing through the sensor. Thereby, the noise response to gases is dependent solely on the properties of the material (resistance) and not on the measurement system.

## Supporting Information Available

SEM images of the nanotube networks of 60, 80, and 90% transparency; long-time photoresponse of the CNT networks of 70 and 90% transparency upon UV 275 nm; resistance of four samples of different transparencies of the CNT network; power spectral density of resistance fluctuations for gas sensing experiments; and time–domain studies for selected concentrations of acetone (PDF)
