# Supplementary material for: Optimum Choice of Randomly Oriented Carbon Nanotube Networks for UV-Assisted Gas Sensing Applications
Source: ACS Sens. 2023 Sep 8;8(9):3547–54. doi: 10.1021/acssensors.3c01185 (PMC10521142; doi:10.1021/acssensors.3c01185)
Supplement: Supplementary file 1 — se3c01185_si_001.pdf [file se3c01185_si_001.pdf]

Supplementary Material

Optimum Choice of Randomly Oriented Carbon Nanotube Networks for UV-assisted Gas Sensing Applications

Katarzyna Drozdowska,[a,*] Adil Rehman,[b] Janusz Smulko,[a] Aleksandra Krajewska,[b] Bartłomiej Stonio,[b,c] Pavlo Sai,[b] Aleksandra Przewłoka,[b,d] Maciej Filipiak,[b,c] Krystian Pavłov,[b,c] Grzegorz Cywiński,[b] Dmitry V. Lyubchenko,[b,e] Sergey Rumyantsev[b]

[a]*Department of Metrology and Optoelectronics, Faculty of Electronics, Telecommunications, and Informatics, Gdańsk University of Technology, G. Narutowicza 11/12, 80-233, Gdańsk, Poland*

[b]*CENTERA Laboratories, Institute of High Pressure Physics PAS, Sokołowska 29/37, 01-142 Warsaw, Poland*

[c]*Centre for Advanced Materials and Technologies CEZAMAT, Warsaw University of Technology, Poleczki 19, 02-822 Warsaw, Poland*

[d]*Institute of Optoelectronics, Military University of Technology, gen. Sylwestra Kaliskiego 2, 00-908 Warsaw, Poland*

[e]*Division of Micro and Nanosystems, KTH Royal Institute of Technology, Malvinas Väg 10, SE-100 44 Stockholm, Sweden*

*Corresponding author – katarzyna.drozdowska@pg.edu.pl

Fig. S1 demonstrates SEM images of nanotube networks of 60%, 80%, and 90% transparency showing randomly oriented structures with different densities.

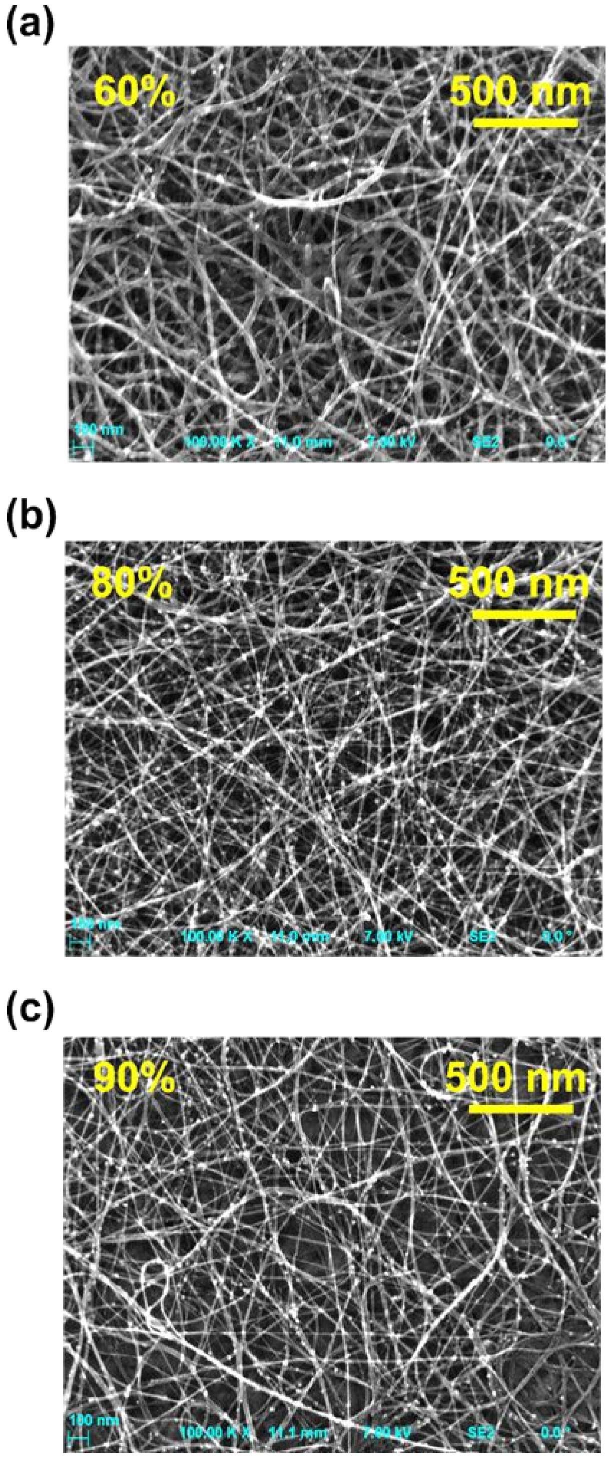

Fig. S1 SEM images of the nanotube networks of **(a)** 60%, **(b)** 80%, and **(c)** 90% transparency.

Fig. S2 depicts long-time (20 minutes) photoresponse for selected carbon nanotube networks (70% and 90% transparency). Photoresponse is presented as the relative change in the sample's resistance compared to resistance in a reference atmosphere (R_0). The higher the transparency, the higher the photoresponse to UV light of 275 nm wavelength. Less dense networks of higher porosity and more specific surface area exposed to UV light tend to interact more significantly with the incoming irradiation. Such an effect can be beneficial for UV-enhanced gas sensing employing DC resistance and low-frequency noise measurements.

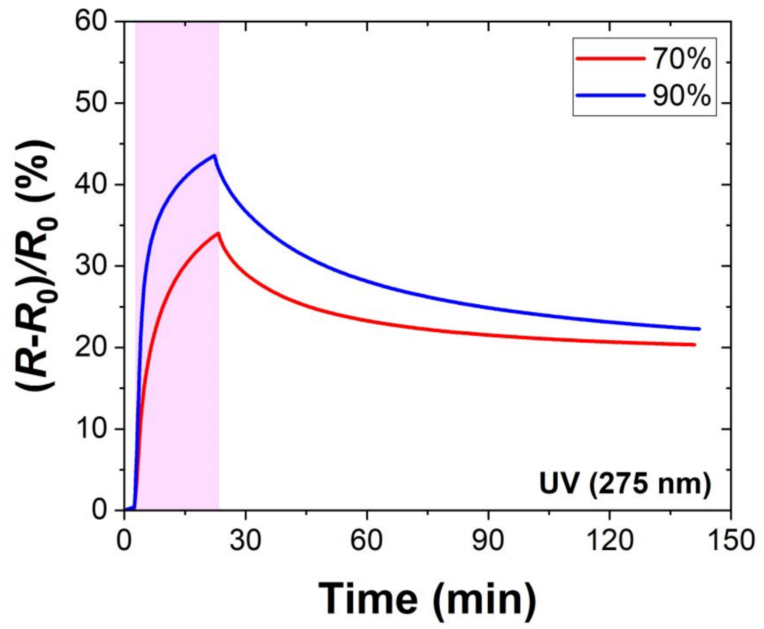

Fig. S2 Long-time photoresponse of the carbon nanotube networks of different transparencies at UV 275 nm showing higher responses of low-dense networks (90%). A shadowed region refers to a 20-min illumination cycle. R_0 denotes baseline resistance at time = 0 min.

Fig. S3 presents the resistance *R* of a few exemplary samples, showing the trend in resistance correlated with the transparency of the layers. Each sample exhibits transparency proportional to the resistance measured for the samples – the higher the network's transparency, the higher the layer's resistance. The dependence between the resistance and the transparency of the carbon nanotube network is linear with R^2 metric = 0.99, where R^2 informs about the adequacy of the linear fitting.

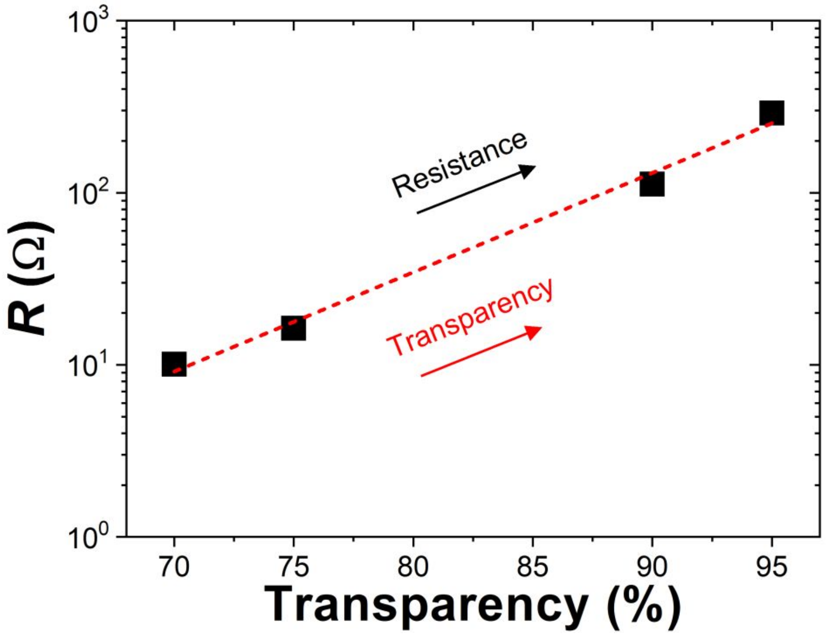

Fig. S3 Resistance of four samples of different transparencies of carbon nanotube network (70%, 75%, 90%, and 95%). The samples of higher transparencies exhibit higher resistance. 70% and 90% samples were chosen for further gas sensing experiments.

Fig. S4 demonstrates low-frequency noise spectra (0.5–1000 Hz) for an exemplary carbon nanotube network of 90% transparency. Both in the dark (Fig. S4a) and under UV light of 275 nm (Fig. S4b), resistance fluctuations increase after ethanol (140 ppm) or acetone (110 ppm) introduction. Comparing 1/*f* noise in synthetic air for dark and UV light conditions, one can notice that the irradiation does not tremendously impact the resistance fluctuations. However, the relative change of normalized power spectral density S_R/R_S^2 increases under UV irradiation during ethanol and acetone detection. In the case of acetone, UV light influences the noise shape drastically, especially for the lowest frequencies (0.5–10 Hz). The noise response is almost eight times higher (149%) than the resistive response (18.7%) under UV light for ethanol and around 22 times higher for acetone (505% of noise response and 23.2% of resistive response), suggesting an increased sensitivity to both gases using fluctuation-enhanced sensing.

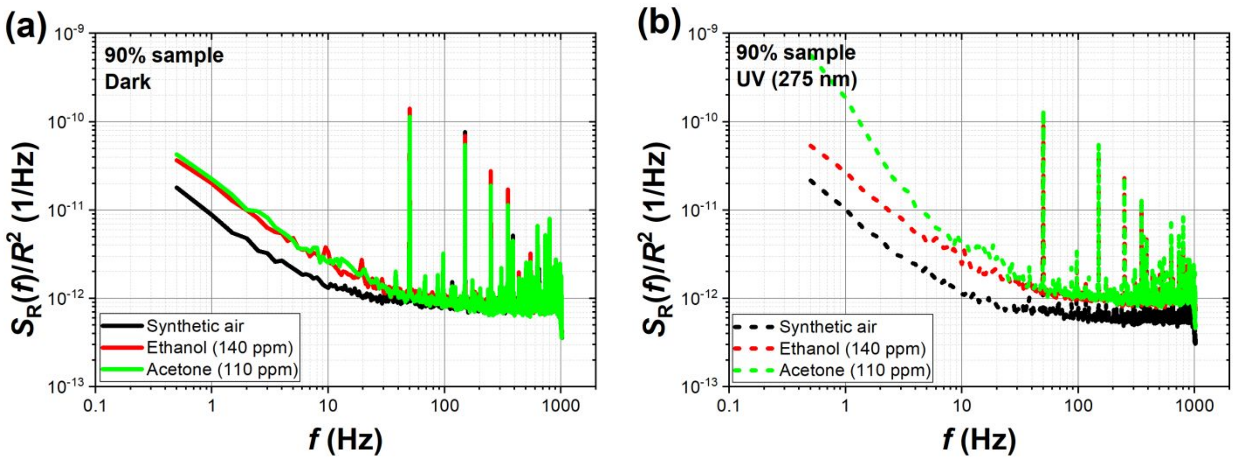

Fig. S4 Power spectral density of resistance fluctuations normalized to DC resistance squared S_R/R_S^2 for the sample of 90% transparency under synthetic air (reference gas) and ethanol (140 ppm) or acetone (110 ppm) **(a)** in the dark, and **(b)** under UV light (275 nm). Noise characteristics were collected for the samples with channel length $L = 200$ µm.

Fig. S5 depicts calibration curves for four cycles of acetone introduction to two samples of different transparencies (high-dense 70% and low-dense 90%). Time-domain responses are accompanied by short-time drift, especially for 70% sample in the dark. The response and recovery rates for dark conditions are poor and almost non-visible, specifically in the third and fourth cycles. The recovery cycles resemble slow saturation with time drift under selected low acetone concentrations in the dark. UV light visibly enhances resistive responses and improves the recovery pace, although 15-minute cycles are not sufficient for the complete return to the baseline resistance.

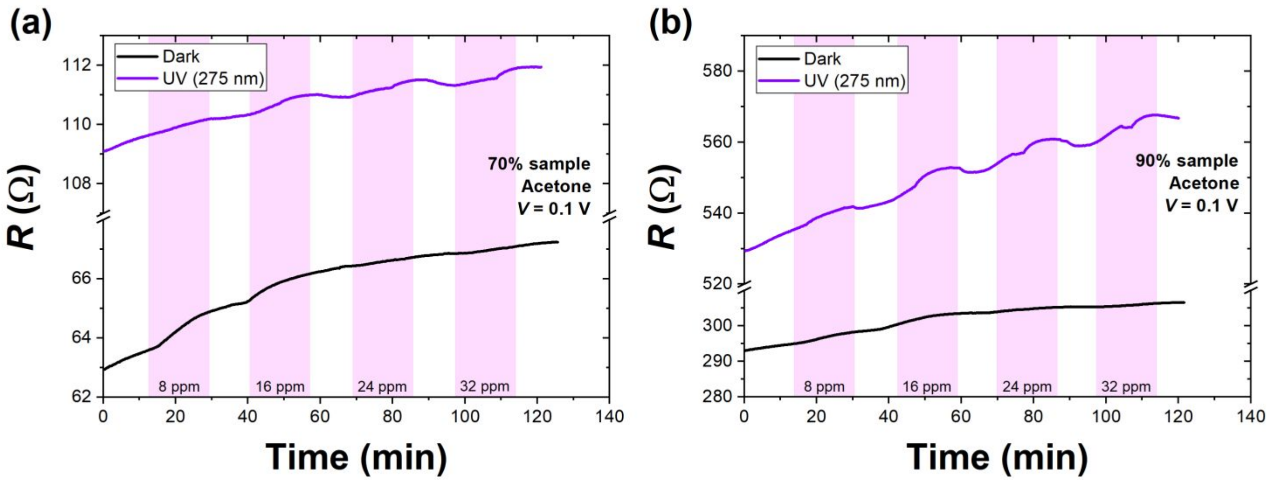

Fig. S5 Time-domain studies for four cycles of acetone introduction (concentrations 8–32 ppm) in the dark and under UV light (275 nm) for carbon nanotube networks sensor of **(a)** 70% transparency and **(b)** 90% transparency. Voltage bias was set to 0.1 V for all time-response measurements.
